# Assessment of Chitosan-Affected Metabolic Response by Peroxisome Proliferator-Activated Receptor Bioluminescent Imaging-Guided Transcriptomic Analysis

**DOI:** 10.1371/journal.pone.0034969

**Published:** 2012-04-04

**Authors:** Chia-Hung Kao, Chien-Yun Hsiang, Tin-Yun Ho

**Affiliations:** 1 Department of Nuclear Medicine, China Medical University Hospital, Taichung, Taiwan; 2 Department of Microbiology, China Medical University, Taichung, Taiwan; 3 Graduate Institute of Chinese Medicine, China Medical University, Taichung, Taiwan; State University of Rio de Janeiro, Biomedical Center, Institute of Biology, Brazil

## Abstract

Chitosan has been widely used in food industry as a weight-loss aid and a cholesterol-lowering agent. Previous studies have shown that chitosan affects metabolic responses and contributes to anti-diabetic, hypocholesteremic, and blood glucose-lowering effects; however, the *in vivo* targeting sites and mechanisms of chitosan remain to be clarified. In this study, we constructed transgenic mice, which carried the luciferase genes driven by peroxisome proliferator-activated receptor (PPAR), a key regulator of fatty acid and glucose metabolism. Bioluminescent imaging of PPAR transgenic mice was applied to report the organs that chitosan acted on, and gene expression profiles of chitosan-targeted organs were further analyzed to elucidate the mechanisms of chitosan. Bioluminescent imaging showed that constitutive PPAR activities were detected in brain and gastrointestinal tract. Administration of chitosan significantly activated the PPAR activities in brain and stomach. Microarray analysis of brain and stomach showed that several pathways involved in lipid and glucose metabolism were regulated by chitosan. Moreover, the expression levels of metabolism-associated genes like apolipoprotein B (apoB) and ghrelin genes were down-regulated by chitosan. In conclusion, these findings suggested the feasibility of PPAR bioluminescent imaging-guided transcriptomic analysis on the evaluation of chitosan-affected metabolic responses *in vivo*. Moreover, we newly identified that downregulated expression of apoB and ghrelin genes were novel mechanisms for chitosan-affected metabolic responses *in vivo*.

## Introduction

Chitosan is a polysaccharide comprising copolymers of glucosamine and *N*-acetylglucosamine. Chitosan has been used as a dietary supplement for decreasing the body weight and lowering the cholesterol level [1]. It is a food additive and can be used as a flocculant and chelating agent for the clarification of beverages [2]. It is also a biodegradable carbohydrate polymer that has been widely used in the tissue engineering, wound healing, biosensers, and drug release [3]–[8]. Previous reports showed that chitosan exhibits anti-diabetic, hypocholesteromic, and blood glucose-lowering effects [9]–[11]. *In vitro* studies also suggested that chitosan inhibits adipogenesis and differentiation of adipocytes [12], [13]. However, the host response to chitosan and the target organs chitosan acted on remain to be clarified.

Peroxisome proliferator-activated receptors (PPARs) are members of the nuclear hormone receptor superfamily. PPAR heterodimerizes the retinoid X receptors and binds to the PPAR responsive element (PPRE) in the promoter region of target genes [14], [15]. So far, three receptor subtypes have been characterized and designated as PPARα, PPARγ, and PPAR-β/δ. PPAR subtypes have distinct tissue localization and physiological activities. PPARα is expressed in brain and liver. It is a central regulator of fatty acid catabolism and glucose metabolism [16]. PPARγ is predominately expressed in adipose tissue, immune system, and gastrointestinal tract. It is the master regulator of adipogenesis, adipocyte and gut epithelial differentiation, lipid storage, and glucose homeostasis [17]. PPAR-β/δ is ubiquitously expressed and plays an important role in regulating energy homeostasis and lipoprotein catabolism [18]. These findings indicate that PPARs are key regulators of lipid and glucose metabolism [19], [20].

We have previously applied nuclear factor-κB bioluminescent imaging-guided transcriptomic analysis to assess host responses to biomaterials, ionizing radiation, and chemotherapy drug *in vivo* [21]–[23]. In this study, we applied PPAR bioluminescent imaging to evaluate the chitosan-affected metabolic responses. Microarray analysis of chitosan-targeted organs was further applied to globally elucidate gene expression profiles of chitosan and to find the novel mechanisms of chitosan. Our data showed that chitosan activated PPAR activities in brain and stomach. Additionally, chitosan regulated several pathways involved in lipid and glucose metabolism, which was in agreement with the well-known hypocholesterolemic and hypoglycemic effects of chitosan.

## Materials and Methods

### Plasmid construction

**Figure 1:**
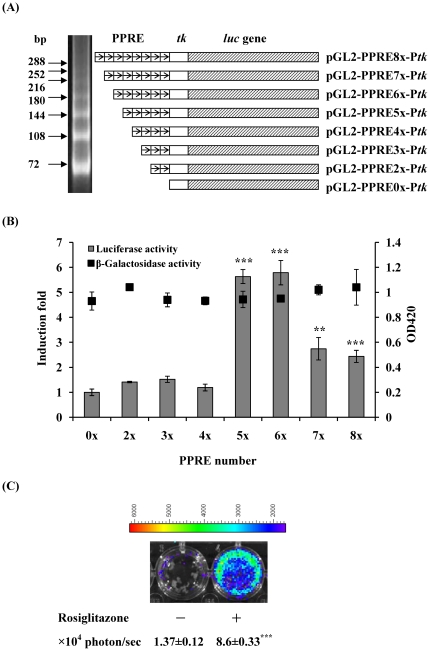
Construction and optimization of PPRE reporter constructs. (A) The schematic diagram of PPRE reporter constructs. Two PPRE oligonucleotides were annealed and ligated to form various tandem repeats of PPRE. The resulting products were analyzed by 8% polyacrylamide gels (left panel). Eight reporter constructs containing various numbers of PPREs were shown on the right. (B) Effect of rosiglitazone on the inducibility of PPRE reporter constructs. HepG2 cells were transiently transfected with PPRE constructs and pcDNA3.1/lacZ DNA, and treated without or with 0.5 µM rosiglitazone. Luciferase and β-galactosidase activities were determined 24 hours later. Luciferase activities are expressed as induction fold, which is presented as comparison with RLU related to untreated cells. β-Galactosidase activities are expressed as OD420. Values are mean ± standard error of three independent assays. **p<0.01, ***p<0.001, compared with untreated cells. (C) *In vitro* imaging. HepG2 cells were transiently transfected with PPRE constructs containing 5 tandem repeats of PPRE and treated without or with 0.5 µM rosiglitazone. Luciferase activity was imaged at 24 h by IVIS system. The color overlay on the image represents the photon/sec emitted from the cells, as indicated by the color scale. Quantification of photon emission from the cells was shown at the bottom. Values are mean ± standard error of three independent assays. ***p<0.001, compared with mock. Photos are representative images.

The 148-bp *Eco*RI/*Xho*I fragment containing the herpes simplex virus *thymidine kinase* (*tk*) promoter from pTKβ (Clontech, Mountain View, CA, USA) was ligated into the *Eco*RI/*Xho*I sites of the pBluescript® II KS (-) vector (Stratagene, La Jolla, CA, USA) to generate pBKS-P*tk*. Two oligonucleotides, PPRE sense (5′-TCGACAGGGGACCAGGACAAAGGTCACGTTCGGGAC-3′) and PPRE antisense (5′-TCGAGTCCCGAACGTGACCTTTGTCCTGGTCCCCTG-3′) containing the PPRE sequence of the acyl-CoA oxidase gene and carrying the *Sal*I and *Xho*I restriction sites at the ends, were annealed and ligated to form two (72 bp), three (108 bp), four (144 bp), five (180 bp), six (216 bp), seven (252 bp), and eight (288 bp) tandem repeats of PPRE. The PPRE tandem repeats were then filled in and inserted into the blunted *Xba*I site of the pBKS-P*tk* vector to generate pBKS-PPRE2x-P*tk*, pBKS-PPRE3x-P*tk*, pBKS-PPRE4x-P*tk*, pBKS-PPRE5x-P*tk*, pBKS-PPRE6x-P*tk*, pBKS-PPRE7x-P*tk*, and pBKS-PPRE8x-P*tk*. The *Sma*I/*Bgl*II fragments containing the *tk* promoter and/or PPRE repeats from aforementioned constructs were then subcloned into the *Sma*I/*Bgl*II sites of the pGL2-Basic vector (Promega, Madison, WI, USA) to generate pGL-PPRE0x-P*tk*, pGL-PPRE2x-P*tk*, pGL-PPRE3x-P*tk*, pGL-PPRE4x-P*tk*, pGL-PPRE5x-P*tk*, pGL-PPRE6x-P*tk*, pGL-PPRE7x-P*tk*, and pGL-PPRE8x-P*tk* (Figure 1(A)).

### Transfection and reporter assays

Human hepatocellular carcinoma HepG2 cells, which were purchased from Bioresource Collection and Research Center (Hsinchu, Taiwan), were maintained in Dulbecco's modified Eagle's medium (Life Technologies, Gaithersburg, MD, USA) supplemented with 10% fetal bovine serum at 37°C. Cells were transfected with PPRE reporter constructs and pcDNA3.1/lacZ DNA (Invitrogen, Carlsbad, CA, USA) by SuperFect® transfection reagent (Qiagen, Valencia, CA, USA). Twenty-four hours later, transfected cells were treated with 0.5 µM rosiglitazone maleate (Alexis, San Diego, CA, USA). Luciferase assay and β-galactosidase assay were performed as described previously [24], [25]. Induction fold was calculated by dividing the relative luciferase unit (RLU) of rosiglitazone-treated cells by the RLU of untreated cells.

### Generation of transgenic mice

Plasmid DNA pGL-PPRE5x-P*tk* was linearized with *Not*I and *Sal*I to generate a 3.1-kb fragment of PPRE transgene for the generation of transgenic mice following pronuclear microinjection of FVB oocytes. Of 18 offspring (F0), six tested positive for PPRE transgene by polymerase chain reaction (PCR) genotyping (primer-P 5′-AACTGCATAAGGCTATGAAGAGATACGCCC-3′ and primer-M 5′-TTAAAACCGGGAGGTAGATGAGATGTGACG-3′). All transgenic mice were crossed with wild-type F1 mice to yield PPRE heterozygous mice with the FVB genetic background.

### Animal experiments

Male transgenic mice (6 to 8 weeks old) were maintained in the room with a 12-h day/12-h night cycle and fed with standard diet *ad libitum*. Mice (*n*=6/group) were subcutaneously injected saline or 0.2 g/kg chitosan. Chitosan oligosaccharide lactate (MW=4000–6000, >90% deacetylation) was purchased from Sigma-Aldrich (St. Louis, MO, USA) and dissolved in DDW. For rosiglitazone treatment, six mice were orally administered 50 mg/kg rosiglitazone. Mice were then imaged for the luciferase activity or sacrificed for microarray analysis at indicated periods.

Mouse experiments were conducted under ethics approval from the China Medical University Animal Ethics Committee (permit number 97-28-N).

### *In vivo* and *ex vivo* imaging of luciferase activity

*In vivo* and *ex vivo* imaging of luciferase activity was performed as described previously [24], [26]. For *in vivo* imaging, mice were anesthetized with isoflurane and injected intraperitoneally 150 mg/kg d-luciferin. Five minutes later, mice were placed in the chamber and imaged for 1 min with the camera set at the highest sensitivity by IVIS Imaging System® 200 Series (Xenogen, Hopkinton, MA, USA). Photons emitted from tissues were quantified using Living Image® software (Xenogen). Signal intensity was quantified as the sum of all detected photon counts from mice and presented as photon/sec. For *ex vivo* imaging, mice were anesthetized and injected with luciferase intraperitoneally. Five minutes later, mice were sacrificed and tissues were rapidly removed. Tissues were placed in the IVIS system and imaged with the same setting used for *in vivo* studies. Signal intensity was quantified as the sum of all detected photon counts per second within the region of interest after subtracting the background luminescence and presented as photon/sec/cm^2^/steradian (photon/sec/cm^2^/sr).

### Microarray analysis

Total RNAs were extracted from brain and stomach as described previously [25]. The RNA sample with a RNA integrity number greater than 7.0 was accepted for microarray analysis.

Microarray analysis was performed as described previously [25]. Briefly, fluorescence-labeled RNA targets were prepared from 5 µg of total RNA using MessageAmp™ aRNA kit (Ambion, Austin, TX, USA) and Cy5 dye (Amersham Pharmacia, Piscataway, NJ, USA). Fluorescent targets were hybridized to the Mouse Whole Genome OneArray™ (Phalanx Biotech Group, Hsinchu, Taiwan) and scanned by an Axon 4000 scanner (Molecular Devices, Sunnyvale, CA, USA). Three replicates from three independent mice were performed. The Cy5 fluorescent intensity of each spot was analyzed by genepix 4.1 software (Molecular Devices). The signal intensity of each spot was corrected by subtracting background signals in the surrounding. We filtered out spots that signal-to-noise ratio was less than 0 or control probes. Spots that passed these criteria were normalized by the R program in the limma package (http://www.r-project.org/). Genes with fold changes ≥2.0 or ≤−2.0 were analyzed by Kyoto Encyclopedia of Genes and Genomes (KEGG) pathway (http://www.genome.ad.jp/kegg/), which is a knowledge base linking a set of genes with a network of interacting molecules in the cells [27]. We used the WebGestalt tool to test significant KEGG pathways. Microarray data are MIAME compliant and the raw data have been deposited in a MIAME compliant database (Gene Expression Omnibus, accession number GSE33565).

### Quantitative real-time PCR (qPCR)

The expression levels of ghrelin, apolipoprotein B (apoB), exportin 4 (xpo4), peptidyl-prolyl *cis*/*trans* isomerase NIMA-interacting 1 (pin1), preproenkephalin 1 (penk1), and peroxiredoxin 2 (prdx2) genes were validated by qPCR. RNA samples were reverse-transcribed for 2 h at 37°C with High Capacity cDNA Reverse Transcription Kit (Applied Biosystems, Foster City, CA, USA). qPCR was performed by using 1 µl of cDNA, 2× SYBR Green PCR Master Mix (Applied Biosystems), and 200 nM of forward and reverse primers. The reaction condition was followed: 10 min at 95°C, and 40 cycles of 15 sec at 95°C, 1 min at 60°C. Each assay was run on an Applied Biosystems 7300 Real-Time PCR system in triplicates. Fold changes were calculated using the comparative C_T_ method. The primer set for each gene is followed: ghrelin forward, 5′-GCTGGAGATCAGGTTCAATGC-3′; ghrelin reverse, 5′-GTCCGTGGTTACTTGTCAGC-3′; apoB forward, 5′-TCTGCCTCTTACTACCCACTG-3′; apoB reverse, 5′-TGTCAACCAAAGACTTGTCCTC-3′; xpo4 forwards, 5′-AGATACCGCAGCTTCCTGAG-3′; xpo4 reverse, 5′- GTGGTCATCTCCGTGTTGTG-3′; pin1 forward, 5′-ATGGCGGACGAGGAGAAG-3′; pin1 reverse, 5′- CGAGACTGGCTGTGCTTC-3′; penk1 forward, 5′-CTTGGGTCCTGCCTCCTG-3′; penk1 reverse, 5′-GCAAGTGGCTCTCATCCTG-3′; prdx2 forward, 5′-CGACCATGCTGAGGACTTC-3′; prdx2 reverse, 5′- TCAACACGCCGTAATTCTGG-3′; glyceraldehyde-3-phosphate dehydrogenase (GAPDH) forward, 5′-ACACCCACTCCTCCACCTTT-3′; GAPDH reverse, 5′-TAGCCAAATTCGTTGTCATACC-3′.

### Statistical analysis

Data were presented as mean ± standard error. Student's *t*-test was used for comparisons between two experiments. A value of *p*<0.05 was considered statistically significant.

## Results

### Optimization of PPRE reporter constructs

Multiple tandem repeats of PPRE were constructed and cloned upstream the *tk* promoter. The resulting PPRE-*tk* constructs were then inserted upstream the luciferase gene and droven the expression of luciferase gene (Figure 1(A)). To test which report constructs were significantly induced by rosiglitazone (a PPARγ agonist), we transiently transfected HepG2 cells with various reporter constructs and treated cells with rosiglitazone. As shown in Figure 1(B), rosiglitazone significantly induced the luciferase activity driven by five, six, seven, or eight tandem repeats of PPRE. The maximal induction was observed in HepG2 cells transfected with five or six tandem repeats of PPRE construct. The β-galactosidase activities were consistent, suggesting that the transfection efficacies were similar in various reporter constructs. Moreover, *in vitro* imaging showed that bioluminescent signal was significantly induced by rosiglitazone in pGL-PPRE5x-P*tk*-transfected HepG2 cells (Figure 1(C)). Ciani et al. constructed the luciferase reporter plasmids containing one, two, three, and five tandem repeats of PPRE, and found that the induced luciferase activity is directly proportional to the number of PPREs present in the promoter region [28]. However, the maximal induction of luciferase activity was observed in HepG2 cells transfected with five or six tandem repeats of PPRE construct in this study. Therefore, we selected the construct containing five tandem repeats of PPRE for further experiment, and these findings suggested that induced luciferase activity might not be proportional to the number of PPREs.

### Characterization of PPRE transgenic mice

Plasmid DNA pGL-PPRE5x-P*tk* was selected for the generation of transgenic mice following pronuclear microinjection of FVB oocytes. Because the transgene contained a luciferase gene driven by PPRE, the luciferase activity reflected the PPAR *trans*-activity.

**Figure 2:**
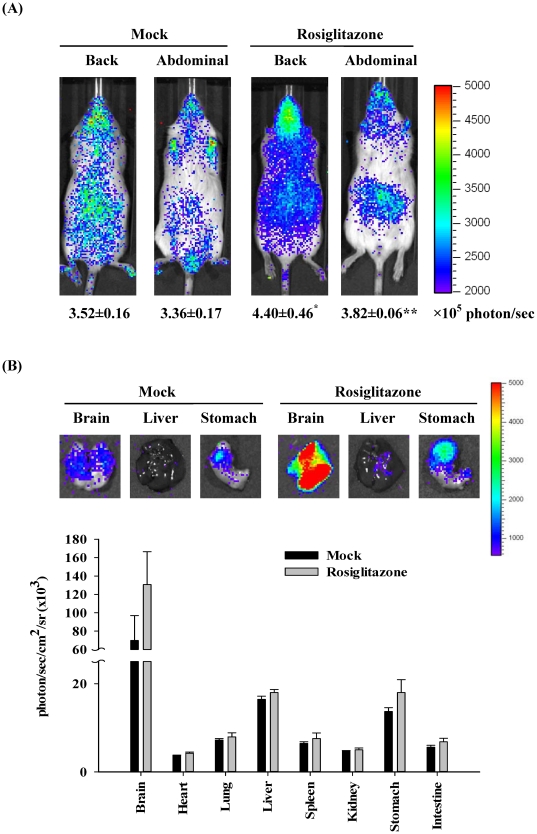
PPAR-dependent bioluminescence in living mice and individual organs. (A) *In vivo* imaging. Transgenic mice were orally administered saline (mock) or 50 mg/kg rosiglitazone and imaged 6 hours later. The color overlay on the image represents the photon/sec emitted from the animals, as indicated by the color scales. Quantification of photon emission from the mice was shown at the bottom. Values are mean ± standard error (*n*=6 per group). *p<0.05, **p<0.01, compared with mock. (B) *Ex vivo* imaging. Transgenic mice were orally administered saline (mock) or 50 mg/kg rosiglitazone. Six hours later, mice were sacrificed and organs were subjected to image. Quantification of photon emission from the organs was shown at the bottom. Values are mean ± standard error (*n*=6 per group). Photos are representative images.

To monitor the constitutive and induced PPAR activity, transgenic mice were treated without or with rosiglitazone and imaged 6 hours later. As shown in Figure 2(A), the diffuse luminescence was detected throughout the body and the intense signals were emitted in the head and abdominal region. Administration of rosiglitazone significantly induced the PPAR-dependent luminescent signals in mice. *Ex vivo* imaging showed that the maximal intensity was observed in the brain, moderate luminescent signals were observed in liver and stomach, and slight intensity was observed in heart, lung, spleen, kidney, and intestine (Figure 2(B)). Administration of rosiglitazone increased the PPAR-dependent luminescent intensity in brain and stomach. These data indicated that endogenous PPAR activities were widely present in most organs and the greater endogenous PPAR activities were observed in brain, liver, and stomach. Moreover, rosiglitazone activated the PPAR activities in the brain and stomach, which was consistent with previous studies [28], suggesting that PPRE transgenic mice could be applied to report the PPAR activity *in vivo*.

### Assessment of PPAR-driven luminescent signal after chitosan administration by bioluminescent imaging

**Figure 3:**
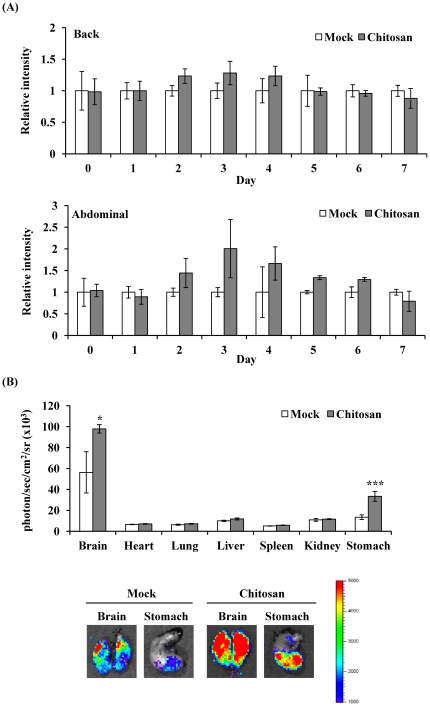
PPAR-dependent bioluminescence in living mice and individual organs after chitosan administration. (A) Time course. Transgenic mice were subcutaneously injected saline (mock) or chitosan, and images at indicated periods. Results are expressed as relative intensity, which is presented as comparison with the luminescent intensity relative to mock. Values are mean ± standard error (*n*=6 per group). (B) *Ex vivo* imaging and quantification of photon emission from individual organs. Transgenic mice were subcutaneously injected saline (mock) or chitosan, and sacrificed 3 days later for organ imaging. Values are mean ± standard error (*n*=6 per group). *p<0.05, ***p<0.001, compared with mock. The color overlay on the image represents the photon/sec emitted from the organs, as indicated by the color scale. Photos are representative images.

Previous studies have shown that chitosan activates the PPAR activity in adipocytes [13]. We further analyzed the *in vivo* PPAR activity after chitosan administration and the targeted organs that chitosan acted on by bioluminescent imaging. Figure 3(A) shows that chitosan gradually increased the luminescence intensity, reached a maximal intensity on 3 d, and gradually decreased the luminescent signals. We further sacrificed mice on 3 d after administration, and *ex vivo* imaging showed that chitosan significantly induced PPAR-dependent bioluminescent signals in brain (1.75 fold) and stomach (2.54 fold) (Figure 3(B)). These findings indicated that administration of chitosan activated PPAR activities in brain and stomach.

### Assessment of chitosan-regulated genes in the brain and stomach by transcriptomic analysis

In order to understand the chitosan-induced biological events in brain and stomach, we extracted RNA samples from brain and stomach on 3 d and performed microarray analysis. In a total of 29,922 genes, the transcripts of 154 and 143 genes in the stomach and brain, respectively, passed the aforementioned criteria (Table S1 and Table S2) and selected for KEGG classification. Tables 1 and 2 show the pathways significantly regulated by chitosan in the stomach and brain, respectively. Five pathways, including oxidative phosphorylation, ribosome, GnRH signaling pathway, tumor necrosis factor-α (TNF-α) signaling pathway and insulin signaling pathway, were affected commonly by chitosan in both organs, and oxidative phosphorylation and ribosome pathways were the top two pathways affected by chitosan. The majority of pathways, including oxidative phosphorylation, pyruvate metabolism, GnRH signaling pathway, glycolysis/gluconeogenesis, benzoate degradation, glycerolipid metabolism, ether lipid metabolism, glutathione metabolism, insulin signaling pathway, alanine and aspartate metabolism and IGF signaling pathway, were associated with lipid and glucose metabolism. These data showed that chitosan might alter several pathways involved in lipid and glucose metabolism in brain and stomach.

**Table 1:** Classification of chitosan-regulated genes in the stomach by KEGG pathways.

KEGG pathway	Stomach	
	Observed (total)^a^	*p* value^b^
Oxidative phosphorylation	38 (108)	8.75×10^−11^
Ribosome	20 (80)	2.17×10^−8^
Cell communication	21 (109)	6.11×10^−5^
IL6 signaling pathway	4 (29)	0.0037
Pyruvate metabolism	11 (37)	0.0040
GnRH signaling pathway	14 (90)	0.0105
TNF-α signaling pathway	3 (34)	0.0105
Glycolysis/Gluconeogenesis	14 (53)	0.0107
Benzoate degradation	2 (7)	0.0215
Glycerolipid metabolism	9 (46)	0.0215
IFNγ signaling pathway	11 (65)	0.0215
LPS signaling pathway	11 (66)	0.0215
Ether lipid metabolism	6 (29)	0.0249
Wnt signaling pathway	14 (144)	0.0325
Glutathione metabolism	9 (41)	0.0363
IL12 signaling pathway	4 (26)	0.0476
Insulin signaling pathway	8 (91)	0.0476

a “Observed" means the number of genes regulated by chitosan in this pathway. “Total" means the total number of genes in this pathway.

b *p*-Value was calculated on WebGestalt web site (http://bioinfo.vanderbilt.edu/webgestalt/login.php) by hypergeometric test.

**Table 2:** Classification of chitosan-regulated genes in the brain by KEGG pathways.

KEGG pathway	Brain	
	Observed (total)^a^	*p* value^b^
Oxidative phosphorylation	25 (108)	5.94×10^−6^
Ribosome	22 (80)	4.49×10^−7^
Amyotrophic lateral sclerosis	3 (17)	0.0117
Gas6 signaling pathway	4 (18)	0.0117
Long-term depression	16 (73)	0.0117
TNF-α signaling pathway	7 (34)	0.0117
PDGF signaling pathway	4 (27)	0.0130
Gap junction	16 (81)	0.0258
Long-term potentiation	14 (64)	0.0258
GnRH signaling pathway	15 (90)	0.0345
Regulation of actin cytoskeleton	22 (193)	0.0345
Insulin signaling pathway	11 (91)	0.0359
Alanine and aspartate metabolism	3 (30)	0.0416
EGF signaling pathway	9 (41)	0.0416
Tight junction	17 (112)	0.0416
Regulation of Ck1/Cdk5	3 (9)	0.0416
IGF signaling pathway	8 (54)	0.0452

a “Observed" means the number of genes regulated by chitosan in this pathway. “Total" means the total number of genes in this pathway.

b *p*-Value was calculated on WebGestalt web site (http://bioinfo.vanderbilt.edu/webgestalt/login.php) by hypergeometric test.

### Verification of the expression levels of chitosan-regulated genes by qPCR

To elucidate which PPAR subtype contributed to the chitosan-affected gene expression profile, we performed Pscan to analyze the PPRE in the promoter regions of chitosan-regulated genes. Pscan is a software that scans promoter sequences of genes with motifs describing the binding specificity of known transcription factors [29]. Over the half of genes contained PPAR-α and/or PPAR-γ responsive elements in their promoter regions, suggesting that the majority of genes were regulated by chitosan via PPAR-α and/or PPAR-γ signaling pathways (data not shown). PPAR-α and PPAR-γ-regulated genes were further validated by qPCR. The expression levels of PPAR-γ-regulated genes, xpo4 and penk1, and PPAR-α-regulated genes, pin1 and prdx2, were upregulated by chitosan in the stomach and brain, which were in agreement with microarray data (Table 3). These data suggested that chitosan regulated gene expression via PPAR-α and/or PPAR-γ signaling pathways.

**Table 3:** Expression levels of ghrelin, apoB, xpo4, pin1, penk1, and prdx2 genes by qPCR.

Sample^a^	Average C_T_ of target	Average C_T_ of GAPDH	ΔC_T_ b	ΔΔC_T_ c	Relative to mock
Ghrelin					
Mock	17.77±0.02	17.56±0.02	0.21±0.03	0.00±0.03	1.00
Chitosan	21.20±0.02	17.57±0.01	3.63±0.02	3.42±0.02	0.09
ApoB					
Mock	26.14±0.02	17.56±0.02	8.58±0.03	0.00±0.03	1.00
Chitosan	29.02±0.05	17.57±0.01	11.45±0.05	2.86±0.05	0.13
Xpo4					
Mock	27.20±0.11	18.56±0.10	8.64±0.15	0.00±0.15	1.00
Chitosan	26.81±0.07	18.93±0.05	7.88±0.09	−0.76±0.09	1.70
Pin1					
Mock	24.11±0.02	18.56±0.10	5.54±0.10	0.00±0.10	1.00
Chitosan	24.04±0.04	18.93±0.05	5.11±0.07	−0.43±0.07	1.35
Penk1					
Mock	25.07±0.04	18.39±0.04	6.68±0.06	0.00±0.06	1.00
Chitosan	22.36±0.06	17.76±0.10	4.60±0.12	−2.08±0.12	4.23
Prdx2					
Mock	22.00±0.00	18.39±0.04	3.61±0.04	0.00±0.04	1.00
Chitosan	20.25±0.07	17.76±0.10	2.49±0.12	−1.12±0.12	2.18

a The RNAs from stomach (ghrelin, apoB, xpo4, and pin1 genes) and brain (penk1 and prdx2 genes) were analyzed for indicated genes by qPCR.

b The ΔC_T_ value is determined by subtracting the average C_T_ of GAPDH from that of target gene. The standard deviation of the difference is calculated from the standard deviations of the target gene and GAPDH.

c The calculation of ΔΔC_T_ involves subtraction by the ΔC_T_ calibrator value. This is a subtraction of an arbitrary constant, so the standard deviation of ΔΔC_T_ is the same as the standard deviation of the ΔC_T_ value.

Ghrelin and apoB are expressed in the stomach and have been shown to be involved in energy and lipid metabolism [30], [31]. Microarray data showed that the expressions of ghrelin and apoB genes were downregulated by chitosan in the stomach, with fold changes of −9.13±0.03 and −5.50±0.25, respectively (Table S1 and Table S2). We further applied qPCR to validate the transcriptional expression levels of these genes. As shown in Table 3, the expression levels of ghrelin and apoB genes were down-regulated by chitosan, which was consistent with the microarray data.

## Discussion

In this study, we found that chitosan significantly activated PPAR activity in brain and stomach. Microarray analysis of brain and stomach further showed that several pathways involved in glucose and lipid metabolism were affected by chitosan. PPARs are ligand-activated nuclear receptors and key regulators of fatty acid and glucose homeostasis [19], [20]. *In vivo* and *ex vivo* imaging showed that maximal luciferase activities were detected in brain and gastrointestinal tract. These data suggested that PPARs were highly expressed in these organs. Previous studies have shown that PPAR activities are activated in brain and gastrointestinal tract, and their activation play important roles in these organs. For examples, in the brain, PPARα plays a major role in acetylcholine biosynthesis and defense against oxidative stress, PPARγ regulates the action of dopamine on the gene transcription, and PPARβ/δ transcriptionally upregulates the acyl-CoA synthetase 2 and mediates the fatty acid utilization [32]. Additionally, the constitutive expression of PPARβ/δ in the gastrointestinal tract is very high compared with other tissues. It plays physiological roles in the homeostatic regulation of intestinal cell proliferation/differentiation and the modulation of inflammation associated with inflammatory bowel disease and colon cancer [33]. Ciani et al (2007) constructed PPAR-luciferase transgenic mice with C57BI/6 genetic background and showed that maximal luciferase activity was detected in the brain and gastrointestinal tract [28]. In this study, we found that PPAR-driven luminescent intensity was strong in the brain and stomach, which was in agreement with previous study. Therefore, these findings suggested that bioluminescent imaging of PPAR transgenic mice was capable of reflecting the real-time PPAR activity in living animals.

Chitosan is a nontoxic, antibacterial, biodegradable, and biocompatible biopolymer. It has been widely used in food and biomaterial industries as weight-loss aids, cholesterol-lowering agents, and medical devices, such as bio-scaffolds for tissue engineering, wound healing products, and haemostatic bandages [1], [3]–[8]. Although chitosan is usually administered by an oral route as a dietary supplement, food additive, or oral drug delivery, chitosan would be degraded by gut microflora or influence the distribution and number of gut microflora by oral administration [1], [2], [34]. Therefore, we administered transgenic mice with chitosan by a parenteral route to avoid the influence of gut microflora. Chitosan induced a maximal intensity on 3 d, and the signal gradually decreased after three days, suggesting that subcutaneous administration of chitosan evoked the PPAR activity, and the induced PPAR activity was decreased to the basal level after 3 days. Administration of chitosan evoked PPAR activations in brain and stomach. These findings suggested that chitosan might affect the biological events in brain and stomach. It has been shown that PPARs play important roles in the pathogenesis of various disorders of central nervous system. For examples, activation of PPARs suppresses inflammation in peripheral macrophages and in models of human autoimmune diseases [35]. Activation of all PPAR isoforms has been found to be protective in murine models of multiple sclerosis, Alzheimer's disease, and Parkinson's diseases [36], [37]. In this study, *ex vivo* imaging showed that chitosan activated the PPAR activity in the brain and the KEGG pathway analysis showed that chitosan significantly regulated the expression of genes involved in amyotrophic lateral sclerosis (*p*=0.012). These findings suggested that chitosan might exhibit the beneficial effect on the neurodegenerative diseases, such as multiple sclerosis, Alzheimer's disease, and Parkinson's diseases.

In addition to brain, chitosan also evoked the PPAR activity in the stomach. KEGG pathway analysis further revealed that the half of chitosan-regulated pathways in the stomach was related to glucose or lipid metabolism. It has been shown that chitosan and its derivatives markedly prevent the time course-related rise of serum glucose levels in diabetic mice [38]. Moreover, chitosan is well known for its hypotriglyceridemic and hypocholesterolemic effects [39], and exhibits anti-obesity and anti-diabetic effects [1], [9]–[11]. Previous studies showed that chitosan and its derivatives may bind to bile salt components and free fatty acids, resulting in the disrupted lipid absorption in the gut and the increased faecal fat excretion [40]. Chitosan also downregulates the adipogenic molecules such as fatty acid binding protein and glucose transporter 4 via PPAR and CCAAT/enhancer-binding protein α pathways, resulting in the inhibited adipogenesis and differentiation of 3T3-L1 adipocytes [12], [13]. Additionally, plasma proteome analysis showed that chitosan increases the level of adiponectin and decreases the levels of obesity-related proteins, such as resistin, retinol-binding protein 4, TNF-α and interleukin-6 (IL-6), contributing to the anti-diabetic and anti-obesity potentials in *ob*/*ob* mice [10]. Our data showed that chitosan significantly regulated the IL-6 and TNF-α signaling pathways in the guts, which were consistent with previous findings. Moreover, we newly identified that chitosan significantly regulated the glucose metabolic pathways, such as glycolysis/gluconeogenesis and insulin signaling pathway, which might contribute to the hypoglycemic effect of chitosan.

Microarray data showed that chitosan downregulated the expressions of apoB and ghrelin genes in the stomach. ApoB, a large amphipathic protein, is mainly expressed in the liver and is present on very-low density lipoproteins (VLDL), intermediate density lipoproteins, and low-density lipoproteins. ApoB is required for the formation of VLDL in the liver. Binding of apoB to the microsomal transport protein results in the incorporation of lipids into the apoB molecule and leads to the formation of VLDL particles [30], [41]. In clinical practice, apoB can be used as a marker to estimate the total number of atherogenic lipoprotein particles [42]. Elevated apoB is a hallmark of several inherited disorders associated with atherosclerosis [43]. However, patients with extremely low levels of apoB seem to be protected against cardiovascular diseases [44]. Because apoB is an essential component of lipoprotein, the down-regulated expression of apoB gene by chitosan might contribute to the hypotriglyceridemic and hypocholesterolemic effects of chitosan. Ghrelin is a peptide hormone mainly produced by the stomach. Ghrelin is a potent stimulator of growth hormone secretion [45]. Moreover, it is the only circulatory hormone that potently enhances the feeding and weight gain, increases the gastrointestinal mobility, and regulates the energy homeostasis [30], [45]. Furthermore, ghrelin-based components may have therapeutic effects in treating malnutrition [31]. Because ghrelin has a great impact on the food intake or body weight, the down-regulated expression of ghrelin gene by chitosan might explain why chitosan exhibited the anti-obestic effect.

In conclusion, we applied PPAR bioluminescent imaging-guided transcriptomic analysis to evaluate the organs that chitosan acted on and to analyze the molecular mechanisms of chitosan in this study. We found that administration of chitosan induced the PPAR-driven bioluminescent signals in brain and stomach. Microarray analysis showed that several pathways associated with lipid and glucose metabolism were regulated by chitosan. Moreover, we newly identified that chitosan may exhibit hypocholestemic and anti-obestic effects via downregulated expression of apoB and ghrelin genes. These findings suggested the feasibility of PPAR bioluminescent imaging-guided transcriptomic analysis on the evaluation of chitosan-affected metabolic responses *in vivo*. Moreover, we newly identified that downregulated expression of apoB and ghrelin genes were the novel mechanisms for chitosan-affected metabolic responses *in vivo*.

## Supporting Information

Table S1 **Expression levels of chitosan-regulated genes in the stomach.**
(PDF)

Table S2 **Expression levels of chitosan-regulated genes in the brain.**
(PDF)
