# Supplementary material for: Assessment of Chitosan-Affected Metabolic Response by Peroxisome Proliferator-Activated Receptor Bioluminescent Imaging-Guided Transcriptomic Analysis
Source: PLoS One. 2012 Apr 4;7(4):e34969. doi: 10.1371/journal.pone.0034969 (PMC3319625; doi:10.1371/journal.pone.0034969)
Supplement: Table S1 — Expression levels of chitosan-regulated genes in the stomach. (PDF) [file pone.0034969.s001.pdf]

Table S1
Expression levels of chitosan-regulated genes[a] in the stomach.

Gene symbol	Gene description	Fold change[b]
Ywhab	Tyrosine 3-monooxygenase/tryptophan 5-monooxygenase activation protein, beta polypeptide	-64.23±0.03
Defb4	Defensin beta 4	-53.75±0.03
Ccdc45	Coiled-coil domain-containing protein 45.	-46.61±0.04
Fbxl14	F-box and leucine-rich repeat protein 14	-43.59±0.04
Col10a1	Procollagen, type X, alpha 1	-18.77±0.09
Psme2	Psme2 protein	-12.70±0.13
Pgc	Progastricsin (pepsinogen C)	-9.45±0.03
Ghrl	Ghrelin	-9.13±0.03
Serpinb8	Serine (or cysteine) peptdiase inhibitor, clade B, member 8	-7.93±0.19
Slc22a6	Solute carrier family 22 (organic anion transporter), member 6	-7.59±0.20
Tff2	Trefoil factor 2 (spasmolytic protein 1)	-7.57±0.07
Itfg1	T-cell immunomodulatory protein precursor	-6.73±0.22
Nos2	Nitric oxide synthase 2, inducible, macrophage	-6.67±0.21
Glod5	Glyoxalase domain-containing protein 5.	-6.51±0.06
Mrpl38	Mitochondrial ribosomal protein L38	-6.36±0.23
Rtp4	Receptor-transporting protein 4 (28 kDa interferon-responsive protein).	-6.07±0.24
Eras	ES cell-expressed Ras	-5.99±0.02
Agr2	Anterior gradient 2 (*Xenopus laevis*)	-5.97±0.03
Apob	Apolipoprotein B	-5.50±0.25
Foxc1	Forkhead box protein C1	-5.48±0.26
Kcne2	Potassium voltage-gated channel, Isk-related subfamily, gene 2	-4.89±0.07
Atp4a	ATPase, H+/K+ exchanging, gastric, alpha polypeptide	-4.86±0.12
Gkn2	Gastrokine-2 precursor	-4.74±0.16
V1rc15	Vomeronasal 1 receptor, C15	-4.68±0.29
Gif	Gastric intrinsic factor	-4.54±0.05
Apoa1	Apolipoprotein A-I	-4.48±0.05
Chia	Acidic mammalian chitinase precursor	-4.48±0.09
Rnf126	Ring finger protein 126	-4.46±0.29
Cga	Glycoprotein hormones, alpha subunit	-4.43±0.30
Clps	Colipase, pancreatic	-4.37±0.09
Lmo4	LIM domain only 4	-4.33±0.05
Mmab	Methylmalonic aciduria (cobalamin deficiency) type B homolog	-4.32±0.32
Alb	Serum albumin precursor.	-4.24±0.05
Serpina1a	Serine (or cysteine) peptidase inhibitor, clade A, member 1a	-4.24±0.08
Ttc28	Tetratricopeptide repeat protein 28 (TPR repeat protein 28).	-4.06±0.33
Gsdma2	Gasdermin 2	-3.99±0.07
Dvl3	Dishevelled 3, dsh homolog (*Drosophila*)	-3.91±0.29
Ms4a1	Membrane-spanning 4-domains, subfamily A, member 1	-3.74±0.33
Sult1c2	Sulfotransferase family, cytosolic, 1C, member 2	-3.72±0.02
Pla2g1b	Phospholipase A2, group IB, pancreas	-3.70±0.21
Rabl3	RAB, member of RAS oncogene family-like 3	-3.63±0.34

Psca	Prostate stem cell antigen	-3.53±0.33
Sst	Somatostatin	-3.53±0.11
Ptp4a1	Protein tyrosine phosphatase 4a1	-3.22±0.37
Gsta2	Glutathione S-transferase, alpha 2	-3.19±0.16
Mal	Myelin and lymphocyte protein, T-cell differentiation protein	-3.15±0.07
Oosp1	Oocyte secreted protein 1	-3.14±0.38
Atp4b	ATPase, H+/K+ exchanging, beta polypeptide	-3.03±0.17
Eif4a2	Eukaryotic translation initiation factor 4A2	-3.00±0.38
Thrsp	Thyroid hormone responsive SPOT14 homolog	-2.94±0.11
Ddx19b	DEAD (Asp-Glu-Ala-Asp) box polypeptide 19b	-2.90±0.40
Pon2	Paraoxonase 2	-2.90±0.40
Cox5a	Cytochrome c oxidase, subunit Va	-2.89±0.23
Osbpl9	Oxysterol binding protein-like 9	-2.88±0.39
Cox7a2	Cytochrome c oxidase, subunit VIIa 2	-2.87±0.12
Glul	Glutamate-ammonia ligase (glutamine synthetase)	-2.81±0.17
Tm4sf5	Transmembrane 4 superfamily member 5	-2.78±0.09
Tff1	Trefoil factor 1	-2.77±0.24
Gcnt3	Beta-1,3-galactosyl-O-glycosyl-glycoprotein beta-1,6-N- acetylglucosaminyltransferase 3	-2.76±0.22
Mei1	Meiosis defective 1	-2.70±0.41
Gsta2	Glutathione S-transferase, alpha 2 (Yc2)	-2.70±0.20
Dapp1	Dual adaptor for phosphotyrosine and 3-phosphoinositides 1	-2.70±0.40
Pgr	Progesterone receptor	-2.69±0.42
Wdr26	WD repeat domain 26	-2.68±0.40
Ptpn9	Protein tyrosine phosphatase, non-receptor type 9	-2.68±0.39
Pla2g12b	Phospholipase A2, group XIIB	-2.59±0.06
Hnrnpa1	Heterogeneous nuclear ribonucleoprotein A1	-2.59±0.38
Dynlt1	Dynein light chain Tctex-type 1	-2.57±0.09
Tra2	Transformer-2 protein homolog	-2.55±0.36
Cox7a1	Polymerase (RNA) II (DNA directed) polypeptide I	-2.53±0.17
Gmppb	Adhesion molecule with Ig like domain 3	-2.52±0.43
Msi2	RNA-binding protein Musashi homolog 2 (Musashi-2).	-2.51±0.40
Scd1	Stearoyl-Coenzyme A desaturase 1	-2.49±0.23
Serpina1e	Serine (or cysteine) peptidase inhibitor, clade A, member 1a	-2.47±0.38
Atp5f1	ATP synthase, H+ transporting, mitochondrial F0 complex, subunit b, isoform 1	-2.44±0.18
S100a11	S100 calcium binding protein A11 (calizzarin)	-2.41±0.22
Ndufs4	NADH dehydrogenase (ubiquinone) Fe-S protein 4	-2.41±0.06
Fkbp3	FK506 binding protein 3	-2.36±0.14
Uqcrb	Ubiquinol-cytochrome c reductase binding protein	-2.35±0.27
Dennd1a	DENN domain-containing protein 1A.	-2.35±0.43
Cox11	COX11 homolog, cytochrome c oxidase assembly protein (yeast)	-2.30±0.11
Csprs	Component of Sp100-rs	-2.30±0.42
Lman1l	Protein ERGIC-53-like precursor	-2.30±0.48
Plac9	Placenta specific 9	-2.29±0.11
Tmem179b	transmembrane protein 179B	-2.26±0.13

Copb1	Coatomer protein complex, subunit beta 1	-2.25±0.19
Mid1	Midline 1	-2.24±0.44
Il17rd	Interleukin 17 receptor D	-2.24±0.43
Axot	Membrane-associated ring finger (C3HC4) 7	-2.23±0.49
Plac8	Placenta-specific 8	-2.21±0.11
Clic6	Chloride intracellular channel 6	-2.20±0.10
Padi2	Peptidyl arginine deiminase, type II	-2.18±0.42
Psp	Parotid secretory protein	-2.16±0.48
Ces2	Carboxylesterase 2	-2.16±0.03
Lpl	Lipoprotein lipase precursor	-2.14±0.06
Ndufb4	NADH dehydrogenase (ubiquinone) 1 beta subcomplex 4	-2.14±0.28
Fundc2	FUN14 domain containing 2	-2.13±0.19
Lgals4	Lectin, galactose binding, soluble 4	-2.13±0.21
Golm1	Golgi membrane protein 1	-2.12±0.06
B2m	Beta-2 microglobulin	-2.10±0.16
Foxa3	Forkhead box A3	-2.09±0.08
Sfn	Stratifin	-2.08±0.38
Ocel1	Occludin/ELL domain-containing protein 1.	-2.08±0.13
Cript	Cysteine-rich PDZ-binding protein	-2.07±0.21
Akr1b8	Aldo-keto reductase family 1, member B8	-2.06±0.13
Cidec	Cell death-inducing DFFA-like effector c	-2.06±0.43
Lsm7	LSM7 homolog, U6 small nuclear RNA associated (*S. cerevisiae*)	-2.06±0.20
Tbca	Tubulin cofactor a	-2.05±0.30
Gstm2	Glutathione S-transferase, mu 1	-2.05±0.27
BC057371	CDNA sequence BC057371	-2.03±0.46
Tmem60	Transmembrane protein 60.	-2.03±0.46
Mgst3	Microsomal glutathione S-transferase 3	-2.03±0.18
Hamp2	Hepcidin-2 precursor	-2.01±0.12
Krt19	Keratin, type I cytoskeletal 19	-2.00±0.24
Prelid2	PRELI domain-containing protein 2	-2.00±0.05
Krtcap3	Keratinocyte associated protein 3	-2.00±0.30
Pla2g12a	Phospholipase A2, group XIIA	-2.00±0.27
Axl	AXL receptor tyrosine kinase	2.00±0.78
Krit1	Krev interaction trapped protein 1	2.00±1.71
Stk4	Serine/threonine kinase 4	2.01±1.63
Ubc	predicted gene, EG216818	2.02±1.54
Prelp	Proline arginine-rich end leucine-rich repeat	2.04±0.72
Col4a2	Procollagen, type IV, alpha 2	2.06±1.07
Itgb1	Integrin beta 1 (fibronectin receptor beta)	2.07±0.26
Kprp	keratinocyte expressed, proline-rich	2.13±1.34
Krt78	Krt78 protein	2.15±1.09
Itm2b	Integral membrane protein 2B	2.16±1.60
Hbp1	High mobility group box transcription factor 1	2.23±0.59
Eef2	Eukaryotic translation elongation factor 2	2.26±0.36

Snph	Syntaphilin	2.29±2.16
Oas1f	2'-5' oligoadenylate synthetase 1F	2.33±2.21
Tpm1	Tropomyosin 1, alpha	2.35±0.51
Krt10	Keratin, type I cytoskeletal 10	2.38±1.56
Rplp1	Ribosomal protein, large, P1	2.42±2.23
Dck	Deoxycytidine kinase	2.67±1.92
Hnrpul1	Heterogeneous nuclear ribonucleoprotein U-like 1	2.67±1.93
Slc10a7	Sodium/bile acid cotransporter 7	3.07±1.95
Armc10	Armadillo repeat-containing protein 10.	3.25±4.09
Fbxo12	F-box and WD-40 domain protein 14	3.56±4.31
Zmynd19	Zinc finger, MYND domain containing 19	3.66±0.65
Lrrn3	Leucine rich repeat protein 3, neuronal	3.75±2.96
Spag9	Sperm associated antigen 9	3.96±2.28
Kif2a	Kinesin-like protein KIF2A.	4.05±2.92
Xpo4	Exportin 4	4.17±5.16
Gnas	GNAS (guanine nucleotide binding protein, alpha stimulating) complex locus	4.37±3.32
Prickle2	Prickle-like protein 2	4.55±6.29
Snrpb	Small nuclear ribonucleoprotein B	4.74±3.93
Pdzk3	PDZ domain containing 2	5.16±3.46
Rshl1	Radial spokehead-like 1	5.30±3.80
Pin1	Protein (peptidyl-prolyl cis/trans isomerase) NIMA-interacting 1	5.61±3.65
Baz1a	Bromodomain adjacent to zinc finger domain protein 1A (Cbp146)	7.52±11.27
Arfgef2	ADP-ribosylation factor guanine nucleotide-exchange factor 2	23.73±39.50
Ralb	V-ral simian leukemia viral oncogene homolog B (ras related)	53.02±90.58
Pitpnb	Phosphatidylinositol transfer protein, beta	192.98±332.62

[a] Genes with fold changes ≥2 or ≤-2 are shown.

[b] Values are mean ± standard error (*n*=3).
