# Supplementary material for: Assessment of Chitosan-Affected Metabolic Response by Peroxisome Proliferator-Activated Receptor Bioluminescent Imaging-Guided Transcriptomic Analysis
Source: PLoS One. 2012 Apr 4;7(4):e34969. doi: 10.1371/journal.pone.0034969 (PMC3319625; doi:10.1371/journal.pone.0034969)
Supplement: Table S2 — Expression levels of chitosan-regulated genes in the brain. (PDF) [file pone.0034969.s002.pdf]

Table S2
Expression levels of chitosan-regulated genes[a] in the brain.

Gene symbol	Gene description	Fold change[b]
Tnfrsf13c	Tumor necrosis factor receptor superfamily, member 13c	-236.40±0.01
Dst	Dystonin	-158.12±0.01
Psd	Pleckstrin and Sec7 domain containing	-110.03±0.02
Aldh1a7	Aldehyde dehydrogenase family 1, subfamily A7	-78.29±0.02
Tmem89	Transmembrane protein 89 precursor.	-67.23±0.03
Mmab	Methylmalonic aciduria (cobalamin deficiency) type B homolog	-48.29±0.04
Zkscan3	Zinc finger with KRAB and SCAN domains 3	-46.71±0.04
Slc25a40	Solute carrier family 25 member 40.	-40.34±0.04
Zfp28	Zinc finger protein 28	-34.87±0.05
Ephb1	Eph receptor B1	-7.90±0.19
Atp6v1e1	VATPase, H+ transporting, lysosomal V1 subunit E1	-7.63±0.20
AU040829	Expressed sequence AU040829	-6.13±0.26
Spag9	Sperm associated antigen 9	-6.06±0.07
Rshl1	Radial spokehead-like 1	-6.06±0.11
Pdzk3	PDZ domain containing 2	-5.71±0.08
Cox7a1	Polymerase (RNA) II (DNA directed) polypeptide I	-5.57±0.26
Dlk2	Delta-like protein 2 precursor	-5.34±0.27
Optn	Optineurin	-5.27±0.27
Pin1	Protein (peptidyl-prolyl cis/trans isomerase) NIMA-interacting 1	-5.07±0.09
Lrrn3	Leucine rich repeat protein 3, neuronal	-4.80±0.13
Gnas	Guanine nucleotide binding protein, alpha stimulating complex locus	-4.60±0.11
Tdpoz1	TD and POZ domain containing 1	-4.40±0.31
Irgc1	Immunity-related GTPase family, cinema 1	-3.84±0.32
Olig3	Oligodendrocyte transcription factor 3	-3.75±0.34
Kif2a	Kinesin-like protein KIF2A.	-3.74±0.10
Fgfr3	Fibroblast growth factor receptor 3	-3.53±0.37
Mpg	N-methylpurine-DNA glycosylase	-3.16±0.36
Speer3	Spermatogenesis associated glutamate (E)-rich protein 3	-3.14±0.37
Uros	Uroporphyrinogen III synthase	-2.99±0.41
Akr1c12	Aldo-keto reductase family 1, member C12	-2.73±0.40
Dlk1	Delta-like 1 homolog (*Drosophila*)	-2.72±0.40
Gabrg1	Gamma-aminobutyric acid (GABA-A) receptor, subunit gamma 1	-2.70±0.43
Hells	Helicase, lymphoid specific	-2.69±0.38
Mmachc	Methylmalonic aciduria and homocystinuria type C homolog.	-2.64±0.38
Mylk	Myosin, light polypeptide kinase	-2.61±0.45
Il1f10	Interleukin 1 family, member 10	-2.59±0.41
Slc10a7	Sodium/bile acid cotransporter 7	-2.58±0.07
Cma1	Chymase precursor	-2.58±0.41
Ccdc125	Coiled-coil domain-containing protein 125.	-2.53±0.41
Fth1	Ferritin heavy chain 1	-2.51±0.26
Calm3	Calmodulin 3	-2.45±0.25

Dck	Deoxycytidine kinase	-2.45±0.34
Glmn	Glomulin, FKBP associated protein	-2.40±0.42
Olfr912	Olfactory receptor 912	-2.37±0.52
Mpv17l	Mpv17 transgene, kidney disease mutant-like	-2.32±0.13
Bcas1	Breast carcinoma amplified sequence 1	-2.30±0.17
Pank4	Pantothenate kinase 4	-2.29±0.54
Dmgdh	Dimethylglycine dehydrogenase precursor	-2.27±0.45
Olfml2a	Olfactomedin-like 2A	-2.23±0.40
Zfp574	Zinc finger protein 574	-2.11±0.40
Nrgn	Neurogranin	-2.10±0.16
Smyd1	SET and MYND domain containing 1	-2.01±0.13
Akr1b3	Aldo-keto reductase family 1, member B3	2.02±1.00
ENSMUSG00000064363	NADH-ubiquinone oxidoreductase chain 4	2.02±1.64
Olfr20	Olfactory receptor 20	2.02±1.68
Cul4b	Cullin 4B	2.02±1.05
Crk	V-crk sarcoma virus CT10 oncogene homolog	2.03±1.52
Grap2	GRB2-related adaptor protein 2	2.03±1.02
Cpne6	Copine VI	2.04±1.07
Rnf38	Ring finger protein 38	2.05±1.83
Ndufa4	NADH dehydrogenase (ubiquinone) 1 alpha subcomplex, 4	2.05±1.86
Ptgir	Prostaglandin I receptor (IP)	2.06±1.93
Zcchc12	Zinc finger, CCHC domain containing 12	2.06±0.49
Amtn	Amelotin precursor.	2.07±1.94
Egfl8	EGF-like domain 8	2.07±1.85
S100a11	S100 calcium binding protein A11 (calizzarin)	2.07±0.85
Rps3	Ribosomal protein S3	2.09±1.39
Cldn19	Claudin 19	2.13±2.01
Cd24a	CD24a antigen	2.13±0.85
Olfr151	Olfactory receptor 151	2.14±2.02
Snap29	Synaptosomal-associated protein	2.15±2.03
Rpusd1	RNA pseudouridylate synthase domain-containing protein 1	2.20±2.07
Pear1	Platelet endothelial aggregation receptor 1 precursor	2.20±1.43
Tas2r120	Taste receptor, type 2, member 120	2.20±2.67
Dysf	Dysferlin	2.24±1.46
Penk1	Preproenkephalin 1 (Opioid hormone)	2.25±0.63
Ttc33	Tetratricopeptide repeat protein 33 (TPR repeat protein 33).	2.29±2.31
Gpx5	Glutathione peroxidase 5	2.30±2.14
Eif2s3y	Eukaryotic translation initiation factor 2 subunit 3, Y-linked	2.30±2.34
Ptk2b	PTK2 protein tyrosine kinase 2 beta	2.31±2.23
Aes	Amino-terminal enhancer of split	2.34±0.66
Aplp1	Amyloid beta (A4) precursor-like protein 1	2.39±0.94
Mbd5	Adaptor protein complex AP-2, mu1	2.41±2.60
Xtrp3s1	X transporter protein 3 similar 1 gene	2.41±1.82
39692	Septin-1	2.43±2.69

Ifi44	Interferon-induced protein 44	2.43±2.47
Fgd6	FYVE, RhoGEF and PH domain containing 6	2.49±2.79
Zfp113	zinc finger protein 113	2.50±2.50
Olfr1	Olfactory receptor 1	2.60±2.80
Snw1	SNW domain-containing protein 1	2.61±2.22
Gdi1	Guanosine diphosphate (GDP) dissociation inhibitor 1	2.63±0.86
Crym	Crystallin, mu	2.67±0.71
Prdx2	Peroxiredoxin 2	2.68±2.87
Olfr836	Olfactory receptor 836	2.68±2.90
Trim14	Tripartite motif-containing 14	2.70±3.02
Olfr284	Olfactory receptor 284	2.88±3.27
Pramel1	Preferentially expressed antigen in melanoma-like 1	2.89±3.35
Sepw1	Selenoprotein W, muscle 1	2.94±0.59
Rcc2	Protein RCC2.	2.96±3.27
Mlf2	Myeloid leukemia factor 2	3.04±1.58
Pcgf6	Polycomb group RING finger protein 6	3.05±3.62
Casq1	Calsequestrin 1	3.06±3.68
Pdcd5	Programmed cell death 5	3.09±3.26
Riok3	RIO kinase 3 (yeast)	3.09±3.84
Cdh13	Cadherin 13	3.13±1.34
Nnat	Neuronatin	3.20±0.58
Tgfbr2	Transforming growth factor, beta receptor II	3.32±3.83
Tgoln1	Trans-Golgi network integral membrane protein 1 precursor (TGN38A).	3.35±4.02
Vps24	Vacuolar protein sorting 24	3.39±4.22
Pds5a	Sister chromatid cohesion protein PDS5 homolog A.	3.41±4.20
Rnf24	Pantothenate kinase 2 (Hallervorden-Spatz syndrome)	3.48±4.32
Eya2	Eyes absent 2 homolog (Drosophila)	3.48±4.24
Mmachc	Methylmalonic aciduria and homocystinuria type C homolog.	3.53±4.33
Znrf1	Zinc and ring finger 1	3.76±4.55
Mrgprd	MAS-related GPR, member D	3.85±4.92
Ube2l3	Ubiquitin-conjugating enzyme E2L 3	4.02±4.71
Exosc6	Exosome complex exonuclease MTR3	4.28±5.62
Gpm6a	Glycoprotein m6a	4.31±5.59
Krt24	Keratin, type I cytoskeletal 24	4.43±6.31
Itpr2	Inositol 1,4,5-trisphosphate receptor type 2	4.67±6.47
Olfr711	Olfactory receptor 711	4.74±6.52
Olfr938	Olfactory receptor 938	4.87±6.72
Ywhab	Tyrosine 3-monooxygenase/tryptophan 5-monooxygenase activation protein, beta polypeptide	4.98±6.49
Il17a	Interleukin-17A precursor	5.04±7.08
Vgll4	Autophagy-related 7	5.06±6.77
Il1rl1l	Transmembrane emp24 domain containing 1	5.15±7.33
Sema3e	Sema domain, immunoglobulin domain (Ig), short basic domain, secreted, 3E	5.21±7.40
Ripk4	Receptor-interacting serine-threonine kinase 4	5.50±7.74
AI593442	Expressed sequence AI593442	5.81±8.45

Cd3g	CD3 antigen, gamma polypeptide	6.04±8.68
Slc39a14	Solute carrier family 39 (zinc transporter), member 14	6.29±9.15
Terf2ip	Telomeric repeat binding factor 2, interacting protein	7.85±11.56
Stmn3	Stathmin-like 3	8.38±11.76
Cbx3	Chromobox homolog 3 (*Drosophila* HP1 gamma)	8.55±13.20
Fbln5	Fibulin 5	9.11±14.09
Olfr1246	Olfactory receptor 1246	9.87±15.50
Vbp1	Von Hippel-Lindau binding protein 1	12.99±20.69
Olfr12	Olfactory receptor 12	13.66±21.91
Pabpn1	Poly(A) binding protein, nuclear 1	17.20±27.49
Oxsm	3-Oxoacyl-[acyl-carrier-protein] synthase, mitochondrial precursor	19.55±32.18
Pkp2	Plakophilin 2	20.50±33.64
G6pc3	Glucose 6 phosphatase, catalytic, 3	23.56±39.14
Trap1	TNF receptor-associated protein 1	69.06±117.34

[a] Genes with fold changes ≥2 or ≤-2 are shown.

[b] Values are mean ± standard error (n=3).
